# Feature Extraction of Ship-Radiated Noise Based on Enhanced Variational Mode Decomposition, Normalized Correlation Coefficient and Permutation Entropy

**DOI:** 10.3390/e22040468

**Published:** 2020-04-20

**Authors:** Dongri Xie, Hamada Esmaiel, Haixin Sun, Jie Qi, Zeyad A. H. Qasem

**Affiliations:** 1 School of Electronic Science and Engineering, Xiamen University, Xiamen 361005, China; 23320171153152@stu.xmu.edu.cn; 2 Department of Information and Communication, School of Informatics, Xiamen University, Xiamen 361005, China; h.esmaiel@aswu.edu.eg; 3 Electrical Engineering Department, Faculty of Engineering, Aswan University, Aswan 81542, Egypt; 4 School of Informatics, Xiamen University, Xiamen 316005, China; hxsun@xmu.edu.cn (H.S.); zeyadqasem@stu.xmu.edu.cn (Z.A.H.Q.)

**Keywords:** ship-radiated noise, feature extraction, enhanced variational mode decomposition, normalized correlation coefficient, permutation entropy

## Abstract

Due to the complexity and variability of underwater acoustic channels, ship-radiated noise (SRN) detected using the passive sonar is prone to be distorted. The entropy-based feature extraction method can improve this situation, to some extent. However, it is impractical to directly extract the entropy feature for the detected SRN signals. In addition, the existing conventional methods have a lack of suitable de-noising processing under the presence of marine environmental noise. To this end, this paper proposes a novel feature extraction method based on enhanced variational mode decomposition (EVMD), normalized correlation coefficient (norCC), permutation entropy (PE), and the particle swarm optimization-based support vector machine (PSO-SVM). Firstly, EVMD is utilized to obtain a group of intrinsic mode functions (IMFs) from the SRN signals. The noise-dominant IMFs are then eliminated by a de-noising processing prior to PE calculation. Next, the correlation coefficient between each signal-dominant IMF and the raw signal and PE of each signal-dominant IMF are calculated, respectively. After this, the norCC is used to weigh the corresponding PE and the sum of these weighted PE is considered as the final feature parameter. Finally, the feature vectors are fed into the PSO-SVM multi-class classifier to classify the SRN samples. The experimental results demonstrate that the recognition rate of the proposed methodology is up to 100%, which is much higher than the currently existing methods. Hence, the method proposed in this paper is more suitable for the feature extraction of SRN signals.

## 1. Introduction

Ships play an important role in both the military and civilian fields. The noise level and directivity of ships vary according to the type of ships. The frequency and propagation distance of the ship-radiated noise (SRN) will also affect the nearby residents to different degrees at different times. As a result, in-depth research on various SRN sources can ensure an uninterrupted life of nearby residents and the reproduction of marine life [1,2,3,4]. The extraction of inherent characteristics of SRN can effectively improve the deficiency of passive sonar and the accuracy of target recognition, making it the most important link in ship classification [5,6].

The initial identification of ships relies only on the judgment of sonar soldiers, but this method is greatly affected by the state, experience, and technology of the soldiers, making the recognition results somewhat subjective. Moreover, in the civil field, related technologies are not sufficiently advanced to meet people’s needs for life. However, with the development of human cognition, a large number of constantly emerging methods have been applied to the classification of underwater acoustic targets, including Fourier transform (FT) [7], short-time Fourier transform (STFT) [8], and wavelet transform (WT) [9]. Nevertheless, these solutions inevitably introduce additional problems. For example, FT can provide sufficient spectral information about the analyzed signal in the frequency domain, but fails to reflect the time-frequency information of the signal. The time-varying characteristics can be reflected by STFT, yet the fixed window length makes it unable to consider both the time domain and frequency domain resolutions. WT inherits and develops the idea of STFT localization, and at the same time overcomes the shortcomings of the window size not changing with the frequency, and can provide a “time-frequency” window that changes with frequency. Its main signal features are gradually multi-scale, refined by telescopic translation operations, achieving time subdivision at a high frequency and frequency subdivision at a low frequency. WT can automatically adapt to the requirements of time-frequency signal analysis, which is an ideal tool for signal processing. However, the presetting of the wavelet basis function and the decomposition layers limits the large-scale practical application of WT [10,11,12].

Entropy is a basic concept in thermodynamics. It can reflect the degree of disorder and the potential dynamics of the system. When the dynamics of the system change, the complexity of the time series changes accordingly. In dynamic theory, entropy is an important indicator of the uncertainty measurement of a nonlinear time series [13]. There are many entropy algorithms that can characterize the randomness of a time series, such as approximate entropy (AE) [14], sample entropy (SE) [15], and permutation entropy [16]. Among them, the permutation entropy (PE) proposed in [17] is widely used in mechanical fault diagnosis [18], agricultural commodity analysis [16], financial sequence analysis [19], and others, due to its fast operation speed and excellent stability. However, PE does not consider that the amplitude of neighboring vectors, and the same ordinal patterns may be different, which leads to a higher estimated value than the actual value. To this end, weighted permutation entropy (WPE) proposed in [20] is superior to PE in identifying the abrupt and stagnant regions of the signal by introducing the weight factor, and can effectively detect the amplitude-encoded information contained in the signal, and resist distortion caused by noise. Therefore, in view of the excellent stability of PE and the strong anti-noise performance of WPE, this article fully integrates the advantages of PE and WPE for the feature extraction of SRN signals.

Despite the remarkable achievements of PE analysis in ship identification [21,22,23], its performance declines in the case of ocean noise. Hence, the de-noising procedure should come before the calculation steps in PE. One way to tackle this issue is via signal decomposition algorithms. The empirical mode decomposition (EMD) method is considered to be a major breakthrough in linear and steady-state spectrum analysis based on FT in 2000. This method is based on the time scale characteristics of the signal itself, without the need to set any basis function. Because of this characteristic, the EMD method can be theoretically applied to the decomposition of any type of signal, giving it an obvious advantage in processing non-stationary and nonlinear data. The key to this method is empirical mode decomposition, which can decompose complex signals into a finite number of intrinsic mode functions (IMFs). Each IMF component that is decomposed contains local characteristic signals at different time scales of the raw signal. Compared with STFT and WT, this method is intuitive, direct, posterior, and adaptive because the basis function is decomposed from the data itself and the decomposition is adaptive based on the local characteristics of the time scale of the signal series. Therefore, once proposed, the EMD method has been rapidly and effectively applied in different engineering fields, such as marine [24], atmospheric [25], and mechanical fault diagnosis [26]. However, mode aliasing has become an inherent defect rooted in EMD, thus limiting its further development. In view of the shortcomings of the EMD method, ensemble empirical mode decomposition (EEMD) [27] proposed a noise-aided data analysis method. The principle of EEMD decomposition is adding the signal with a uniformly distributed white noise background, and signal regions of different scales are automatically mapped to the appropriate scale related to the background white noise. The noise in the EEMD is eliminated when the overall mean of the sufficient tests is used. The overall mean will eventually be considered the true result. With more and more tests, the additional noise is eliminated, and the only lasting and stable part is the signal itself. However, EEMD still has shortcomings. Firstly, similar to EMD, the decomposition results of EEMD method also include residual components. Secondly, due to the randomness of Gaussian white noise, the results of EEMD are different between each decomposition time. That makes EEMD lack a solid mathematical foundation to be widely accepted. In contrast to EMD and EEMD, variational mode decomposition (VMD) [28] can adaptively decompose the signal into several quasi-orthogonal IMFs without generating residual components. VMD determines the frequency center and bandwidth of each component by iteratively searching for the optimal solution of the variational model in the process of obtaining the decomposed components, so that it can adaptively achieve the frequency domain segmentation of the signal and execute the effective separation of the components.

EMD, EEMD, and VMD can decompose signals into a group of intrinsic mode functions (IMFs) that can reflect the local characteristics of the raw signal from different time scales. Due to the marine environmental noise, not every IMF can fully characterize the raw signal. Some of the obtained components are signal-dominant IMFs, while the others belong to noise or noise-dominant IMFs and should be removed before PE extraction. A growing number of studies have attempted to select signal-dominant IMFs with satisfactory results. In [21], EMD-EIMF-PE, the signal-dominant IMF by EMD, was selected according to the energy criterion, whose PE was regarded as a characteristic parameter. In [29], the IMF with the highest energy was screened out to calculate its multi-scale permutation entropy (MPE), which achieved a recognition rate of 94%. In [30], the VMD decomposition of the SRN was performed, the IMF with the smallest fluctuation-based dispersion entropy (FDE) difference from the raw signal was then selected to characterize the raw signal, reaching a recognition rate of 97.5%. In the study in [31], IMFs were first obtained by the complementary ensemble empirical mode decomposition with adaptive noise (CEEMDAN) decomposition of SRN signals. The normalized mutual information was then applied to remove the noise-dominant IMFs and the multiscale improved permutation entropy (MIPE) weighted by the normalized mutual information served as the feature vector, achieving a higher recognition rate at different signal-to-noise ratios. Compared with the existing methods, although both [29] VMD-EIMF-MPE and [30] VMD-SMIF-FDE have achieved high recognition rates, some problems still exist that need to be solved urgently. First of all, in [29,30], the mode number *K* of VMD was calculated referring to the decomposition results of EMD, which will undoubtedly affect the accuracy of VMD decomposition. Moreover, in both studies, only one signal-dominant IMF was used for feature extraction without considering the others. In this way, some important information embedded in the raw signal will be lost. Finally, in [30], the optimum IMF selection method lacks reasonable mathematical derivation and relies on experience.

Following that purpose, in this paper, a new feature extraction technique for SRN is proposed based on enhanced VMD (EVMD), normalized correlation coefficient (norCC), and PE. In order to calculate the mode number *K* of VMD, the VMD mode number range is first set according to the EEMD decomposition results and the variance of the IMF center frequency at each decomposition result is calculated. The mode number, maximizing the variance, is used as an optimum value of the VMD. Subsequently, EVMD decomposition is performed on the three types of SRN samples. The WPE of each IMF by EVMD and variance of $\left({\mathrm{WPE}}_{1}{\sim \mathrm{WPE}}_{i},\;i=1,2,\ldots ,K\right)$ are calculated, thus *K* variance values can be obtained totally. Allowing the IMF index corresponding to the maximum variance to be *k* (k≤K), IMF_1_~IMF*_k_* can be regarded as the signal-dominant IMFs. After that, the correlation coefficient (CC) between each signal-dominant IMF and the raw signal and PE of each signal-dominant IMF are calculated, respectively. After this, the norCC is utilized to weigh the corresponding PE, and the sum of the weighted PE is used as the final feature parameters.

The structure of the paper is presented as follows: the background is described in Section 2. Section 3 introduces the basic steps of the proposed method. Section 4 applies the proposed method to the simulated signal. In Section 5, the new method is utilized for the feature extraction of measured data. Finally, the conclusion of the paper is presented in Section 6.

## 2. Background

This section will present the state of the art for feature extraction algorithms, such as VMD, norCC, PE, and WPE.

### 2.1. Variational Mode Decomposition 

Variational mode decomposition (VMD) [28] defines the IMF as a function of instantaneous amplitude *Am_k_* (*t*) and phase *Ph_k_* (*t*) of an amplitude-modulated-frequency-modulated (AM-FM) signal given as:

(1) $$I_{k}\left(t\right)=Am_{k}\left(t\right)\cos\left(Ph_{k}\left(t\right)\right)$$

In VMD algorithm, the raw signal *s*(*t*) is decomposed into several IMFs in order to find the variational problem. The operation of the VMD can be expressed as follows:

(2) $$\begin{array}{c} \min_{\left\{I_{k},f_{k}\right\}}\left\{{\sum_{k}\left\|\partial_{t}\left[\left(\delta \left(t\right)+\frac{j}{\pi t}\right)*I_{k}\left(t\right)\right]e^{-j2\pi f_{k}t}\right\|}_{2}^{2}\right\}\\ s.t.\sum_{k}I_{k}=s(t) \end{array}$$

where $\partial_{t}$, $\delta_{t}$ and *f_k_* is the partial derivative, impulse function and center frequency of the *I_k_*(*t*), respectively. The constrained variation problem of the VMD in (2) was addressed using the quadratic penalty term and the Lagrange multipliers as follows:

(3) $$\begin{array}{ll} L(\left\{I_{k}\right\},\left\{f_{k}\right\},\lambda ) & =\alpha {\sum_{k}\left\|\partial_{t}\left[\left(\partial_{t}+\frac{j}{\pi t}\right)*I_{k}\left(t\right)\right]e^{-j2\pi f_{k}t}\right\|}_{2}^{2}\\ & +{\left\|s(t)-\sum_{k}I_{k}(t)\right\|}_{2}^{2}+\langle \lambda (t),s(t)-\sum_{k}I_{k}(t)\rangle \end{array}$$

where α and λ denotes the penalty factor and Lagrange multiplier, respectively. $I_{k}^{n+1}$, $f_{k}^{n+1}$ and $\lambda^{n+1}$ are updated as follows:

(4) $${\hat{I}}_{k}^{n+1}(f)=\frac{\hat{s}(f)-\sum_{i\neq k}{\hat{I}}_{i}(f)+\frac{\hat{\lambda }(f)}{2}}{1+2\alpha {\left(f-f_{k}\right)}^{2}}$$

(5) $$f_{k}^{n+1}=\frac{\int_{0}^{\infty }2\pi f{\left|{\hat{I}}_{k}(f)\right|}^{2}df}{\int_{0}^{\infty }{\left|{\hat{I}}_{k}(f)\right|}^{2}df}$$

(6) $${\hat{\lambda }}^{n+1}(f)={\hat{\lambda }}^{n}(f)+\varepsilon (\hat{s}(f)-\sum_{k}{\hat{I}}_{k}^{n+1}(f))$$

where *ε* represents the updated parameter. The VMD stop condition is given by:

(7) $${\sum_{k}\left\|{\hat{I}}_{k}^{n+1}-{\hat{I}}_{k}^{n}\right\|}_{2}^{2}/{\left\|{\hat{I}}_{k}^{n}\right\|}_{2}^{2}<a$$

where *a* is the convergence accuracy. 

### 2.2. Correlation Coefficient and Normalized Correlation Coefficient

The correlation coefficient (CC) of two discrete random variables X and Y can be expressed as:

(8) $$\rho_{X,Y}=\frac{\sum \left(X-\bar{X}\right)\left(Y-\bar{Y}\right)}{\sqrt{\sum {\left(X-\bar{X}\right)}^{2}\sum {\left(Y-\bar{Y}\right)}^{2}}}$$

where $\bar{X}=\sum_{i=1}^{N}x_{i}/N$, $\bar{Y}=\sum_{i=1}^{N}y_{i}/N$, and N denotes the data length of time series.

CC can measure the degree of correlation between variables. If two variables are completely uncorrelated, CC = 0; If two variables are completely correlated, CC = 1. After performing the de-noising process, IMFs that do not contribute significantly to classification will be removed, the remaining ones are called as signal-dominant IMFs. In order to weigh the PE (presented in Section 2.3) values of the signal-dominant IMFs, the normalized correlation coefficient (norCC) corresponding to each signal-dominant IMF is defined as:

(9) $$norCC_{i}=\frac{\rho_{IMF_{i},\;S}}{\sum_{j=1}^{k}\rho_{IMF_{j},\;S}},\;k\leq K$$

where *k* is the number of signal-dominant IMFs, *K* is the mode number of VMD, and *S* denotes the raw signal.

### 2.3. Permutation Entropy

Permutation entropy (PE) [17] can not only measure the randomness of time series, but also detect the dynamic changes of that time series. The PE only compares neighboring values and has fast operation speed. Literature has shown how PE is effective in noise observation and dynamic change detection of time series. The detailed calculation steps of PE can be summarized as follows:

For the given time sequences {x(i),i=1,2,⋯,N}, it can be reconstructed as:

(10) $$X_{i}=\left\{x(i),x(i+\tau ),\ldots ,x(i+(m-1)\tau )\right\},i=1,2,\ldots ,N-\left(m-1\right)\tau$$

where *m* is the embedding dimension and τ is the time delay.

Rearrange *X_i_* elements in an increasing order as:

(11) $$x(i+(j_{1}-1)\tau )\leq x(i+(j_{2}-1)\tau )\leq \cdots \leq x(i+(j_{m}-1)\tau )$$

and in case of two of the rearranged elements are equal it will be:

(12) $$x(i+(j_{1}-1)\tau )=x(i+(j_{2}-1)\tau )$$

then the new order can be denoted as:

(13) $$x(i+(j_{1}-1)\tau )\leq x(i+(j_{2}-1)\tau )(j_{1}\leq j_{2})$$

By this way, a group of symbols can be obtained as:

(14) $$S(g)=(j_{1},j_{2},\cdots ,j_{m})$$

where *S*(*g*) represents one of the *m*! symbol sequences in phase space, g=1,2,…,k,k≤m!.

If the probability distribution of the symbol sequence is $P_{1},P_{2},\cdots ,P_{k}$, the normalized PE for convergence is defined as follows:

(15) $$H_{p}(m)=-{\left(\ln m!\right)}^{-1}\sum_{g=1}^{k}P_{g}\ln P_{g}$$

The value of PE ranges is from 0 to 1. The larger PE value means more random time series; while the smaller PE value indicates less random time series.

### 2.4. Weighted Permutation Entropy

The main PE shortcoming is inconsideration of the neighboring vectors having the same ordinal patterns but with different amplitudes. The weighted permutation entropy (WPE) [20] was proposed to overcome the PE drawbacks. In the WPE, for a given embedding dimension *m* and time delay τ, first, the weight $w_{j}$ of the neighboring vectors *X_i_* is calculated as:

(16) $$w_{j}={\sum_{k=1}^{m}\left[x_{j+\left(k-1\right)\tau }-{\bar{X}}_{j}^{m,\tau }\right]}^{2}$$

(17) $${\bar{X}}_{j}^{m,\tau }=\frac{1}{m}\sum_{k=1}^{m}x_{j+\left(k+1\right)\tau }$$

Then, the weighted relative frequency is calculated as:

(18) $$p_{w}\left(\pi_{i}^{m,\tau }\right)=\frac{\sum j\leq N^{1_{u:\mathrm{type}\left(u\right)=\pi_{i}}\left({\bar{X}}_{j}^{m,\tau }\right)w_{j}}}{\sum j\leq N^{1_{u:\mathrm{type}\left(u\right)\in \Pi }\left({\bar{X}}_{j}^{m,\tau }\right)w_{j}}}$$

At the end, the WPE definition is described as follows:

(19) $$H_{w}\left(m,\tau \right)=-\sum_{i:\pi_{i}^{m,\tau }\in \Pi }p_{w}\left(\pi_{i}^{m,\tau }\right)\ln\left(p_{w}\left(\pi_{i}^{m,\tau }\right)\right)$$

## 3. The Proposed Feature Extraction Method

**Figure 1:**
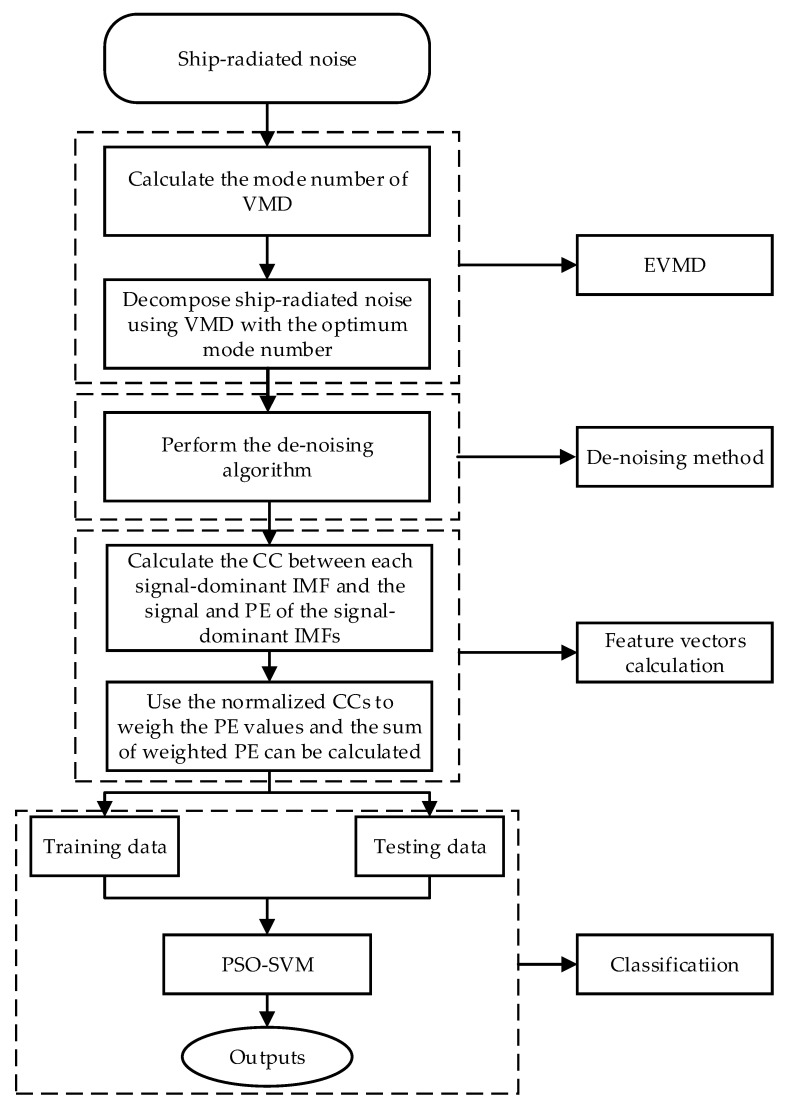
The flowchart of the proposed method.

The VMD decomposition method has been widely used in time–frequency analysis in recent years for underwater target recognition. No studies have applied norCC to underwater target recognition. In the direction of recognizing the underwater target, this paper proposes combining the EVMD, norCC, and PE for a multi-stage feature extraction method. The described flowchart for the proposed feature extraction method is shown in Figure 1. The operation steps for the proposed method can be summarized as follows:

(1) The mode number *K* of VMD is first calculated using the variance of the IMF center frequency to improve the decomposition performance of VMD.
(2) The VMD algorithm will be applied on the measured SRN data using the optimum mode number *K* obtained in the first step.
(3) Calculate the WPE of each IMF obtained by EVMD and the variance of $\left({\mathrm{WPE}}_{1}{\sim \mathrm{WPE}}_{i},\;i=1,2,\ldots ,K\right)$, thus totally *K* variance values can be obtained. Allowing the IMF index to correspond to the maximum variance *k* (k≤K), the IMF_1_~IMF_k_ can be regarded as the signal-dominant IMFs retained and the remaining ones will be removed.
(4) Calculate the CC between each signal-dominant IMF and the raw signal and PE values of the signal-dominant IMFs.
(5) Use the norCCs to weigh the PE values and the sum of the weighted PE can be calculated. Namely, $SWPE=\sum_{i=1}^{k}norCC_{i}\cdot PE_{i}$.
(6) The obtained feature vectors are randomly divided into two groups, first one is the training data for training the SVM classifier and the second one is testing data for classification.
(7) Finally, the testing data are fed into the PSO-SVM multi-class classifier for underwater acoustic target recognition and classification.

## 4. Simulated Signals Analysis

### 4.1. Analysis of Simulated Signals Using EVMD

In order to validate the effectiveness of the EVMD algorithm, EMD, EEMD, and EVMD are respectively performed to decompose the simulated signals. The expression of the simulated signals is expressed as follows:

(20) $$\left\{ \begin{array}{l} f_{1}(t)=\cos(10\pi t)\\ f_{2}(t)=\cos(60\pi t)\\ f_{3}(t)=\cos(110\pi t)\\ f_{4}(t)=\cos(1600\pi t)\cdot e^{-10^{3}{\left(t-2.5\right)}^{2}}\\ f(t)=f_{1}(t)+f_{2}(t)+f_{3}(t)+f_{4}(t)+\eta \end{array}\right.$$

where the data length is set to be 5000 with a sampling frequency of 1 kHz and η is the Gaussian white noise with CN(0,0.5).

**Figure 2:**
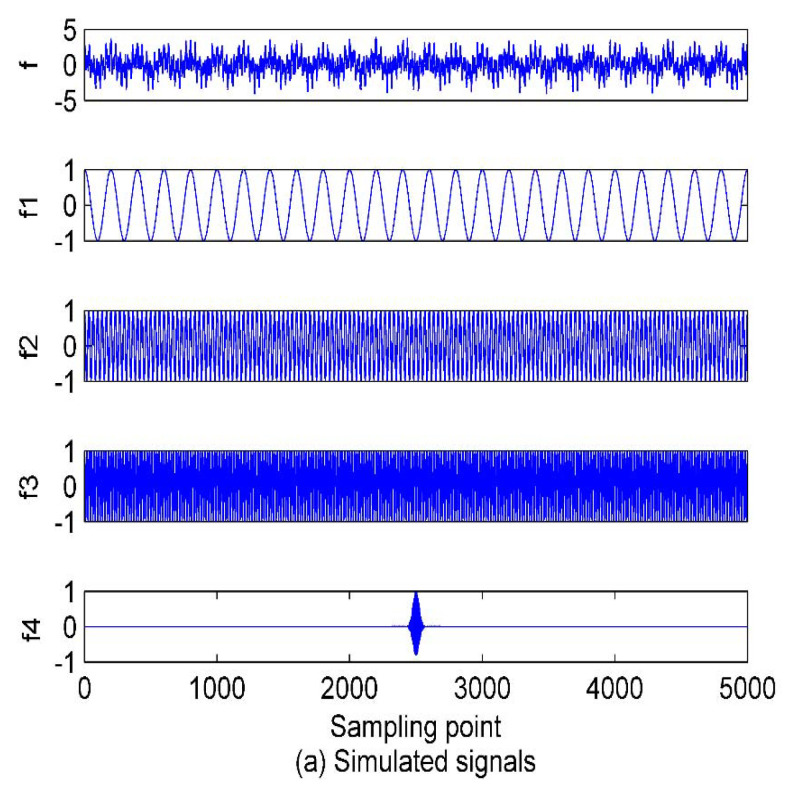

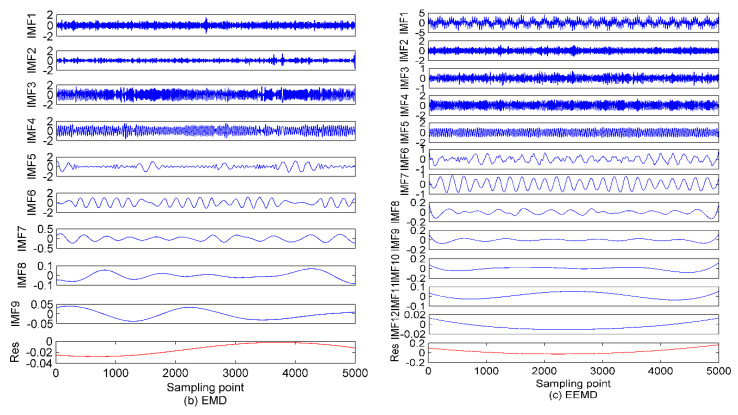
The time domain waveforms of simulated signals and decomposition results of empirical mode decomposition (EMD) and ensemble empirical mode decomposition (EEMD); (**a**) the raw simulated signals; (**b**) EMD method; (**c**) EEMD method.

As in [28], before performing the VMD algorithm, the optimum mode number should be adjusted for the significant influence on the VMD decomposition performance. The other parameters are always set as constants. Namely, the data-fidelity constraint is α=2000, the convergence tolerance level is $tol=1e^{-7}$, and the update modes of center frequency are init=0,1,2 for center frequencies iterated with 0, uniform distribution, or randomly. If the mode number *K* is too large, over-decomposition will be inevitably introduced, which means one or more spurious components will be produced. However, if the mode number *K* is too small, an under-decomposition will occur and the IMFs with useful information will be discarded during the decomposition process, and some important information characterizing the analyzed signal will be lost. Hence, a properly chosen *K* is critical to the VMD method. The related studies in both [29] and [30] referred to the decomposition results of EMD, which will undoubtedly affect the VMD decomposition accuracy. In addition, by combining the simulated results of EMD, EEMD, and VMD in [29,30], it can be observed that the mode number of VMD is not greater than EEMD, and EMD and EEMD in this case are first implemented to decompose the simulated signals above. Figure 2 shows the decomposition results and the time domain waveforms of the analyzed signals.

**Figure 3:**
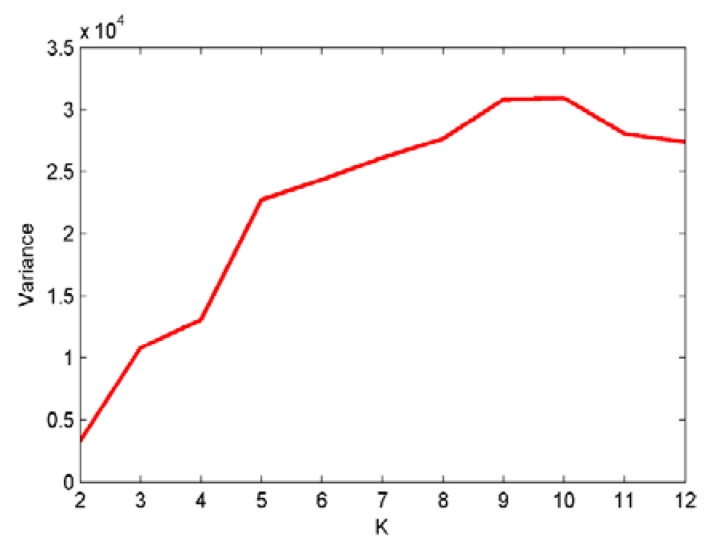
The curve for the variance of IMF center frequency with *K* by variational mode decomposition (VMD).

**Figure 4:**
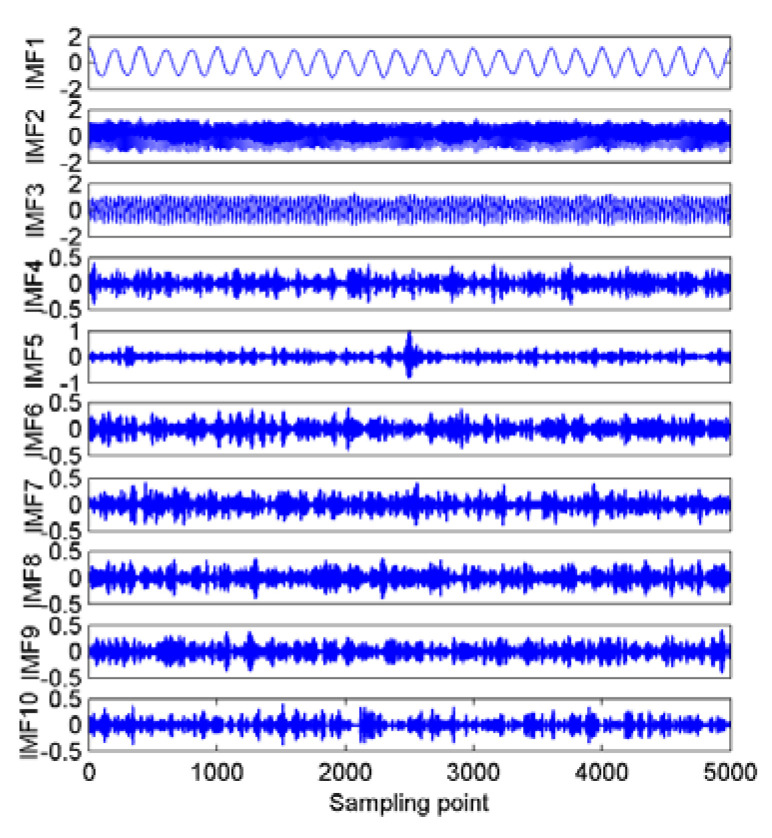
The enhanced variational mode decomposition (EVMD) results for the simulated signals.

As shown in Figure 2, after the decomposition of the simulated signals by EMD, nine IMFs and one residual component are obtained. In case of the EEMD method, 12 IMFs and one residual component are separated in an orderly manner. Hence, we can set the mode number range to be (2~12), and calculate the variance of the center frequency of the obtained IMFs after each decomposition. Theoretically speaking, the optimum mode number *K* is the one which maximizes the variance, as the maximum variance means the most significant difference between the IMF center frequency. The variance curve of the IMF center frequency versus mode number *K* after the simulated signals analyzed by VMD is shown in Figure 3. As shown in Figure 3, the maximum variance corresponding to the mode number is 10, hence, *K* = 10 is considered as an optimum value and for *K* greater than it, the variance starts to decrease. This includes the insignificant difference between IMF center frequency and the occurrence of over-decomposition in VMD method. The decomposition results of the simulated signals decomposed by VMD using the obtained *K* is shown in Figure 4. Table 1 lists the decomposition layers corresponding to the distribution of the IMF center frequency. We can observe in Table 1 that when *K* = (2~8), the components of the simulated signal are not completely separated; when *K* is increased to be nine or 10, the simulated signals are completely separated. In addition, *K* = (11~12) can correctly realize the separation of the simulated signals, but more spurious components are generated. Based on the decomposition results, the proposed method of using the variance of IMF center frequency in this paper is significantly effective in locking the mode number of VMD. In order to further validate for the effective use of the method in the VMD algorithm, the correlation coefficients between corresponding IMF and simulated signals are calculated. The results are listed in Table 2.

Combining Figure 2 and Figure 4 with Table 2, for the EMD and EEMD methods, the IMFs corresponding to the simulated signals *f*_1_, *f*_2_, and *f*_3_ can be deduced properly, but the IMF corresponding to *f*_4_ is submerged by noise and cannot be identified. This is due to the mode aliasing rooted in EMD and EEMD. In contrast, EVMD can roughly reproduce the IMF corresponding to *f*_4_, and its correlation coefficient with *f*_4_ is 0.4123, this indicates a moderate correlation between the two signals. However, the corresponding EMD and EEMD values are only 0.1100 and 0.1337, respectively, which denotes a very weak correlation or uncorrelation between the two signals. The separated IMFs by EVMD have the largest correlation coefficients with the simulated signals with the best decomposition performance. Furthermore, in the EMD and EEMD methods, the first few IMFs are high-frequency components, and they have relatively strong correlations with the raw signals, so they can be considered as the signal-dominant IMFs. These IMFs contain almost all the information of the analyzed signals. In contrast, the last few IMFs of the EMD and EEMD contain poor information of the raw signals, especially for the residual component. These IMFs are almost uncorrelated with the raw signals and can be considered as noise-dominant components or noise. The same situation occurs in the EVMD algorithm, the information and correlation related to the raw signal in the IMFs are gradually distributed from the first to the last IMFs. The first five IMFs are the most important group compared to the remaining ones. These first five low-frequency IMFs belong to the signal-dominant components, and the remaining high-frequency ones are considered as the noise-dominant components or noise and should be removed before PE extraction.

### 4.2. PE Properties Analysis

**Figure 5:**
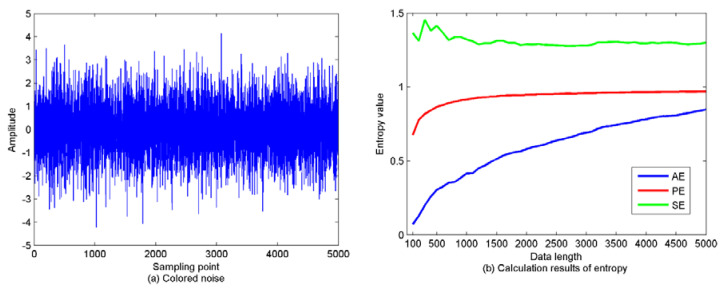
The time-domain waveforms of colored noise and calculation results of approximate entropy (AE), permutation entropy (PE), and the sample entropy (SE). (**a**) the colored noise series; (**b**) the calculation results of AE, PE, and SE.

The ideal white noise is only a theoretical abstraction, which is difficult to achieve physically, and there is no such noise in reality. The noise contained in the measurement data of the engineering practice is often colored. Colored noise can be seen as the output of a linear link driven by a white noise sequence. Therefore, to explore the influence of data length on PE, a colored noise series is generated with a length of 5000. As recommended in [17,32], the time delay and embedding dimension are set as one and six, respectively. We then calculate PE, AE, and SE with 100 as the step size, thus there are 50 values for each kind of entropy. The time-domain waveforms and calculation results are shown in Figure 5.

As shown in Figure 5, as data length increases, the trends of AE and SE fluctuate more severely, while the trend fluctuations of PE are relatively gentle. Therefore, PE is more suitable for feature extraction with better stability than AE and SE.

## 5. Feature Extraction of Ship-Radiated Noise Based on EVMD-norCC-PE

### 5.1. The EVMD Decomposition of Ship-Radiated Noise

**Figure 6:**
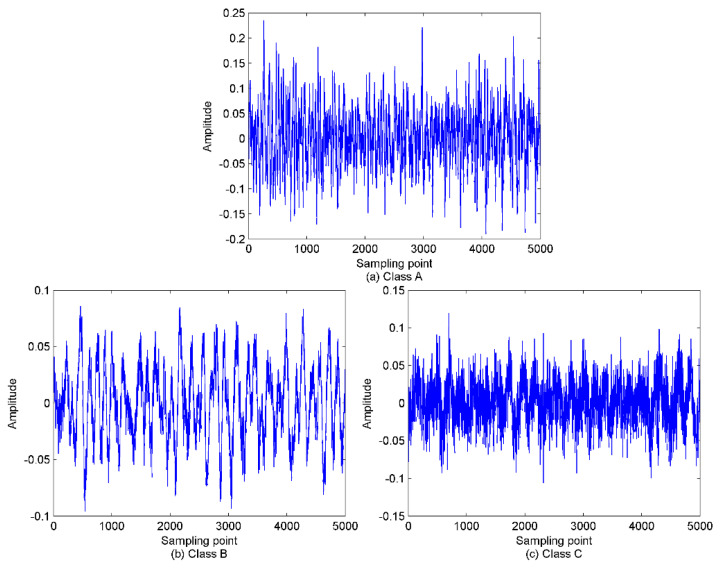
The time-domain waveforms for the three types of normalized ship-radiated noise (SRN). (**a**) Class A signal; (**b**) Class B signal; (**c**) Class C signal.

**Figure 7:**
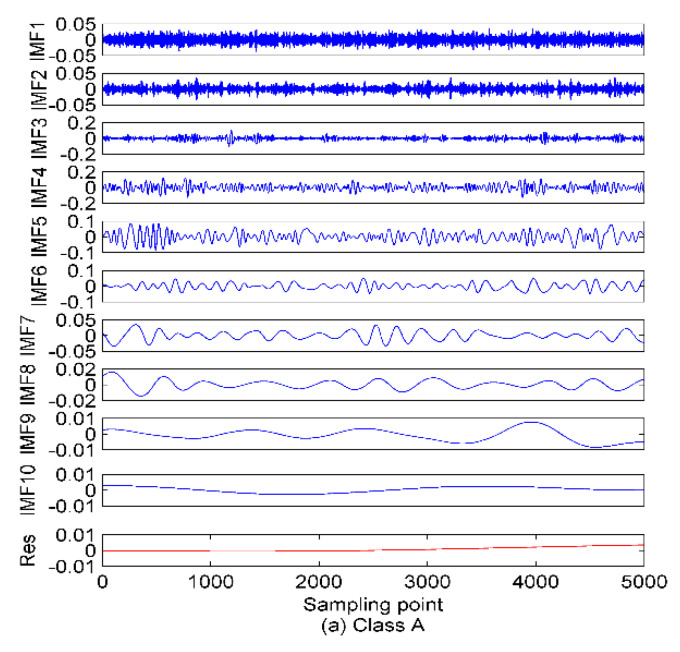

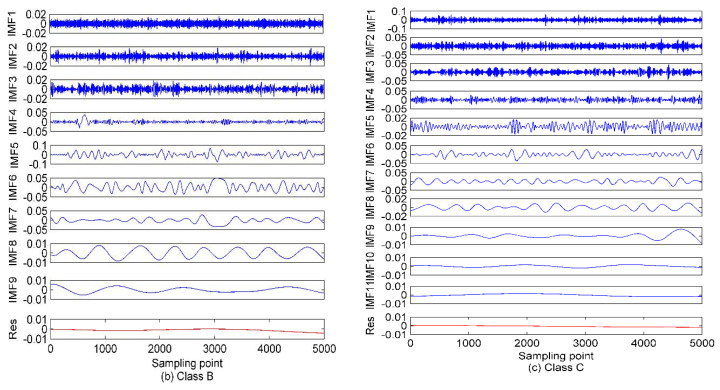
The EMD of the SRN signals. (**a**) Class A; (**b**) Class B; (**b**) Class C.

**Figure 8:**
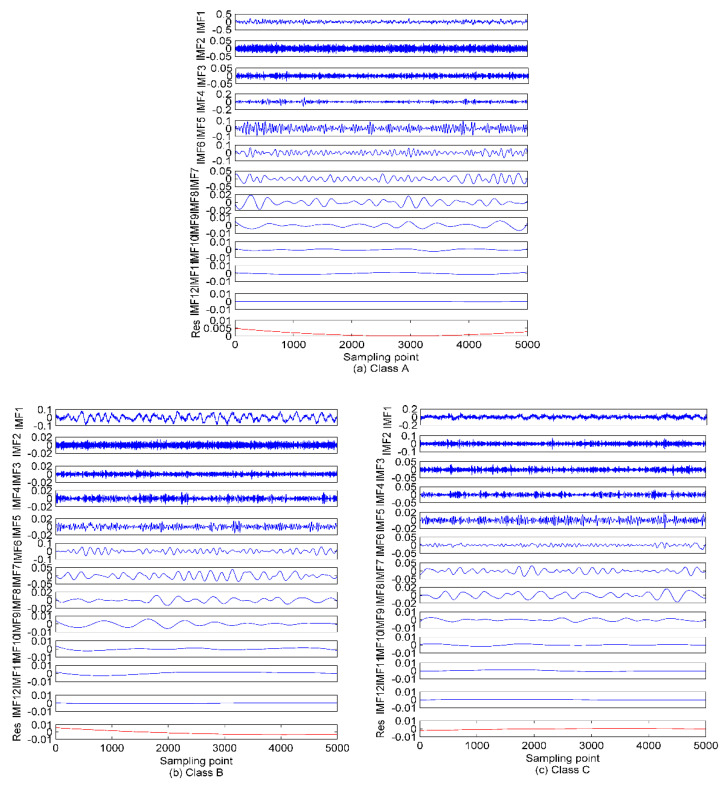
The EEMD of the SRN signals. (**a**) Class A; (**b**) Class B; (**c**) Class C.

In this paper, the feature extraction method is evaluated for SRN by applying the proposed scheme to three types of SRN data randomly selected from a real measured dataset used in [33]. The SRN measured data used in this paper were recorded on the Atlantic coast in northwestern Spain (42°14′ N, 008°43.4′ W), at a depth of 10 m. The sampling frequency and data length for this measured data are set to 52.734 kHz and 5000, respectively. The selected three SNR samples are named in this paper as class A, B, and C, respectively. The time-domain waveforms of these SNR signals are shown in Figure 6. As explained in the first step of the proposed method, the mode number *K* will be calculated. The EMD and EEMD algorithms are first employed to decompose the SRN signals to calculate the mode number for VMD. The decomposition results of this step are shown in Figure 7 and Figure 8.

**Figure 9:**
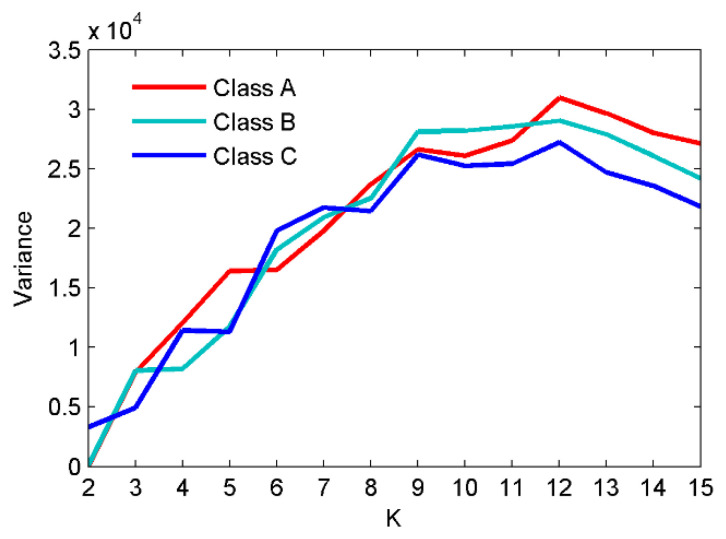
The variance analysis for the SRN signals.

**Figure 10:**
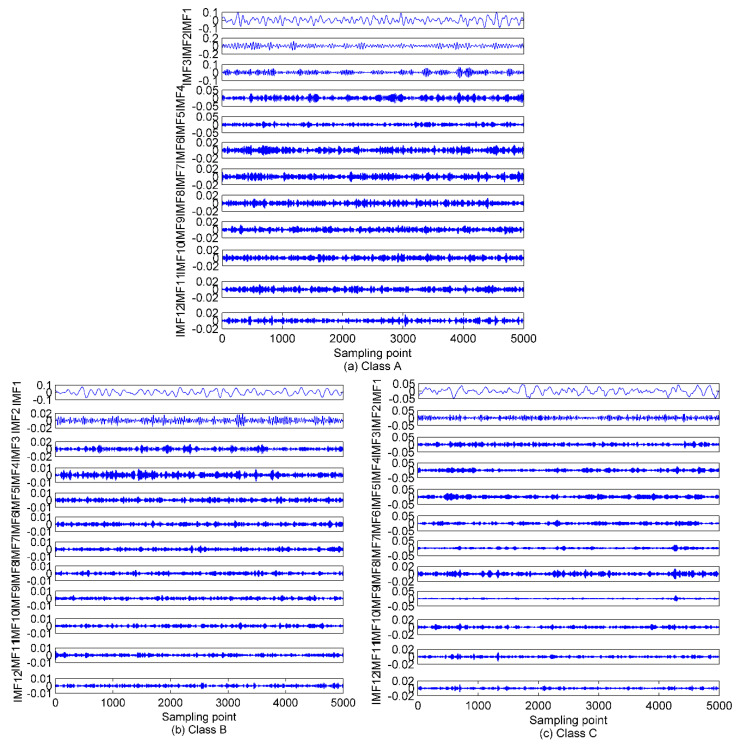
The EVMD of the SRN signals. (**a**) Class A; (**b**) Class B; (**c**) Class C.

As seen in Figure 8, 12 IMFs and one residual component are obtained after EEMD of each type of SRN. For the sake of observation, *K* is set as (2~15) to calculate the variance of the IMF center frequency in VMD. The results of the variance analysis for the SRN signals are presented in Figure 9. The results show the optimal *K* is 12, and for *K* larger than it the variance sharply decreases, and that means the occurrence of over-decomposition in VMD. Figure 10 shows the decomposition results by EVMD.

### 5.2. The De-Noising Processing 

**Figure 11:**
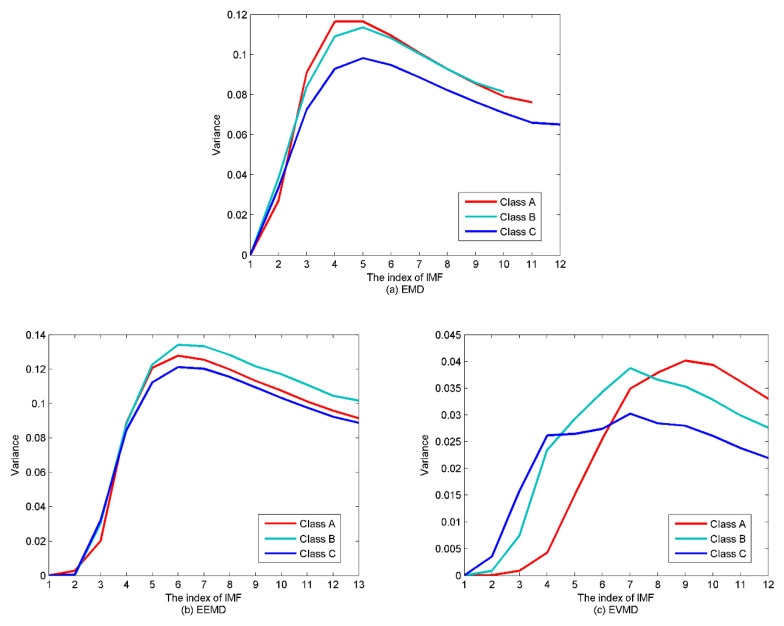
The de-noising method for the algorithms. (**a**) EMD; (**b**) EEMD; (**c**) EVMD.

Combining Figure 7, Figure 8 and Figure 10 with the simulated results of Section 4.1, after the three types of SRN processed by EMD and EEMD, the first few IMFs are high-frequency components, which carry rich information about the analyzed signal, while the last few are low-frequency components containing poor information. Furthermore, for the EMD and EEMD algorithms, the fluctuations of the IMFs from top to bottom become smoother and smoother, indicating the decreasing complexity of these IMFs. For example, in the EMD and EEMD algorithms, the fluctuations of IMF_10_~Res for the three types of samples are extremely small and cannot effectively characterize the raw signal. They should be removed before feature extraction. In the EVMD algorithm, the first few IMFs carry rich amplitude information related to the raw signal, and the difference between the last few high-frequency components or noise gradually decreases, and they should not be included in the entropy feature analysis. In summary, the signals detected by passive sonar are inevitably contaminated by the marine environmental noise. This marine noise can greatly hinder the classification of ships. To handle this issue, a de-noising processing should be preprocessed first before the feature extraction. WPE has good anti-noise ability, and the variance is also a good candidate for indicating the data fluctuation, as a larger variance means high data fluctuation and vice versa. Therefore, the de-noising method proposed in this paper (presented in Section 3) is applied to select the signal-dominant IMFs for EMD, EEMD, and EVMD. The de-noising results are presented in Figure 11. The main important IMFs (signal-dominant IMFs) containing the most important information about the SNR signals are listed in Table 3. 

### 5.3. Classification of Ship-Radiated Noise 

**Figure 12:**
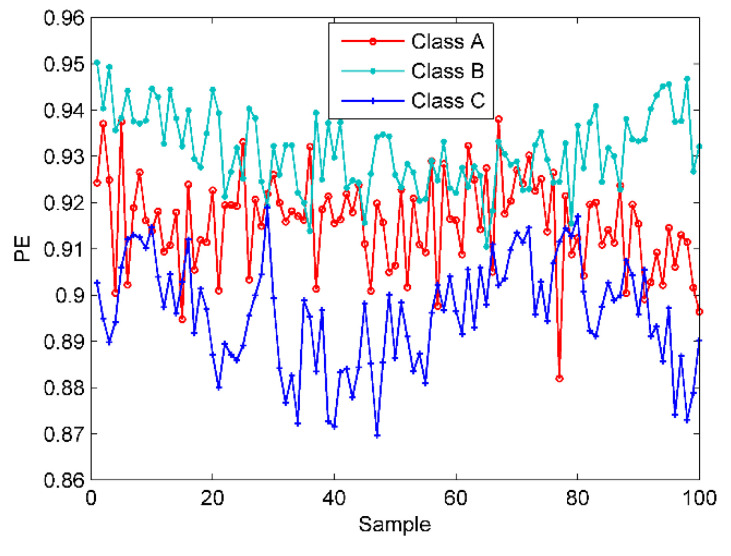
The PE distribution of the three types of SRN samples.

In this section, the proposed technique is applied to deal with the three types of SRN. In this SRN samples, there are 100 randomly selected samples in each class by 300 in total. In order to verify the effectiveness of the proposed method, we calculate the PE values of these samples. Figure 12 is the PE distribution of the three types of samples.

**Figure 13:**
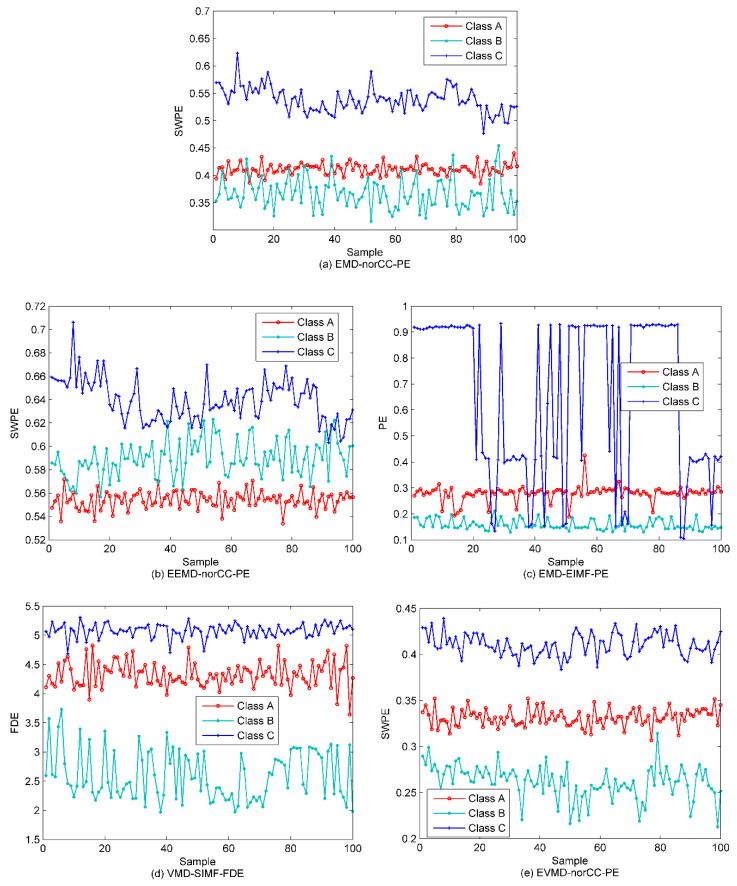
The scatter plots under different algorithms. (**a**) EMD-norCC-PE; (**b**) EEMD-norCC-PE; (**c**) EMD-EIMF-PE; (**d**) VMD-SIMF-FDE; (**e**) EVMD-norCC-PE.

As shown in Figure 12, due to the marine environmental noise, most of the three types of samples are crossed together and cannot be distinguished at all. The signal decomposition algorithms should be used in this case due to the mixed samples. For a fair comparison, the scatter plots of EMD-norCC-PE, EEMD-norCC-PE, EMD-EIMF-PE [21], VMD-SIMF-FDE [30] and EVMD-norCC-PE are presented in Figure 13.

We can observe from Figure 13 that, concerning the EMD-norCC-PE algorithm, the three types of samples can be generally recognized, especially for category C, while there are still a large number of samples of class A and B that cross together. However, compared with the PE-based method, the discriminating ability was significantly improved. For EEMD-norCC-PE, class A and class B samples can be better separated, which produces better performance than EMD--norCC-PE. The EMD-EIMF-PE can generally differentiate the three types of samples, but several samples in class C intersect with the others and cannot be separated well. In contrast, the VMD-SIMF-FDE method can clearly distinguish these samples with fewer cross-samples, but the distance between the classes A and C is still small. Compared with the above algorithms, the proposed EVMD-norCC-PE can separate these SRN samples well, and the distance between classes increases significantly. While the EVMD-norCC-PE differs little from VMD-SIMF-PE in distinguishing these samples, owing to the inaccurate presentation of the scatter plots, it can only provide a qualitative analysis rather than quantitative comparison of algorithms such that the PSO-SVM multi-class classifier, in this case, is introduced for a more detailed analysis for the performance of these algorithms. 

After this, 60 samples are randomly selected from each class to be used as training the classifier and the remaining ones are left to be used for testing the performance. These testing data are fed into the PSO-SVM multi-class classifier to realize the classification of ship targets. The classification outputs together with the computing time are shown in Table 4. As illustrated in Table 4, the PE-based method has the most misclassified samples with a 70% recognition rate that falls far short of the need for classification. In comparison, the accuracy of the proposed method is 100%, which is much higher than the other algorithms. The experimental results show how the proposed method in this paper is clearly superior to the currently existing methods. As far as the computing time is concerned, the computing time of all the algorithms is relatively long due to the low configuration of the computer. The computation time of EVMD-norCC-PE is shorter than that of EEMD-norCC-PE, but longer than that of other algorithms. When the proposed algorithm is applied in practice, the computing time can be shortened by improving the hardware configuration.

## 6. Conclusions

In order to extract features of ship-radiated noise, a novel feature extraction approach based on EVMD, norCC, and PE is proposed in this paper. The EVMD tackled the issue of existing VMD methods in terms of mode number such that it is utilized to decompose the ship-radiated noise signals in this paper for the first time. Compared with AE and SE, PE has better stability and faster operation speed. The proposed feature extraction approach fully integrates the merits of both the EVMD and PE methods. In marked contrast to the currently existing methods for feature extraction of ship-radiated noise, the EVMD-norCC-PE scheme eliminates the noise-dominant components hidden in the IMFs obtained by EVMD using the proposed de-noising processing prior to PE extraction. Moreover, it utilizes norCC to weigh the PE results with each signal-dominant IMF covered. The reliability of the proposed algorithm is verified by the experiments on ship classification. The experimental results indicate that the recognition rate of the proposed method is as high as 100%, which is much higher than other methods. Therefore, the proposed method is more applicable to feature extraction for underwater acoustic signals in practice. 

The proposed enhanced VMD method conquers the mode number difficulty in VMD without consideration of other parameters. In future work, the optimization of both the mode number and quadratic penalty term will be addressed to further improve the decomposition accuracy of EVMD.

## Figures and Tables

**Table 1:** The decomposition layers corresponding to the distribution of intrinsic mode function (IMF) center frequency by VMD.

K	Center Frequency/Hz											
2	30.00	199.06										
3	7.73	52.69	361.73									
4	7.69	52.70	203.13	363.84								
5	7.68	52.72	200.82	320.38	436.90							
6	7.53	52.72	116.38	208.54	356.33	445.45						
7	7.50	52.73	113.67	200.28	277.92	362.95	448.28					
8	7.48	52.74	112.02	196.09	257.40	316.82	373.65	452.45				
9	5.04	55.11	30.00	126.71	202.06	275.88	353.44	408.78	467.51			
10	5.04	55.10	30.01	124.02	197.93	256.75	312.74	365.13	424.43	474.04		
11	5.04	55.08	30.00	112.05	158.93	206.26	263.24	316.16	366.31	452.29	474.42	
12	5.04	55.08	30.00	111.49	157.37	204.59	259.33	310.70	358.79	400.70	440.86	481.01

**Table 2:** Correlation coefficients between corresponding IMF and simulated signals.

	EMD	EEMD	EVMD
*f*_1_	IMF_6_: 0.9294	IMF_7_: 0.9575	IMF_1_: 0.9936
*f*_2_	IMF_4_: 0.8845	IMF_5_: 0.9822	IMF_3_: 0.9925
*f*_3_	IMF_3_: 0.8665	IMF_4_: 0.9525	IMF_2_: 0.9896
*f*_4_	IMF_2_: 0.1100	IMF_2_: 0.1337	IMF_5_: 0.4123

**Table 3:** The distribution of signal-dominant IMFs using the proposed de-noising method for EMD, EEMD and EVMD.

Class	EMD	EEMD	EVMD
A	IMF_1_~IMF_5_	IMF_1_~IMF_6_	IMF_1_~IMF_9_
B	IMF_1_~IMF_5_	IMF_1_~IMF_6_	IMF_1_~IMF_7_
C	IMF_1_~IMF_5_	IMF_1_~IMF_6_	IMF_1_~IMF_7_

**Table 4:** The outputs of classification under different algorithms.

Method	Number of Misclassified Samples			Accuracy Rate (%)	Computing Time (Second)
	Class A	Class B	Class C		
PE	19	9	8	70	1205.346847
EMD-norCC-PE	2	9	0	90.8333	2044.39669
EEMD-norCC-PE	2	3	3	93.3333	17,778.779946
EMD-EIMF-PE	5	0	8	89.1667	1342.94618
VMD-SIMF-FDE	2	0	0	98.3333	8344.834518
EVMD-norCC-PE	0	0	0	100	16,472.39683
